# Public Support for Democracy in the United States Has Declined Generationally

**DOI:** 10.1093/poq/nfad039

**Published:** 2023-09-15

**Authors:** Christopher Claassen, Pedro C Magalhães

**Affiliations:** Senior Lecturer, School of Social and Political Sciences, University of Glasgow, Glasgow, UK; Research Professor, Institute of Social Sciences (ICS), University of Lisbon, Lisbon, Portugal

## Abstract

Support for democracy in the United States, once thought to be solid, has now been shown to be somewhat shaky. One of the most concerning aspects of this declining attachment to democracy is a marked age gap, with younger Americans less supportive of democracy than their older compatriots. Using age-period-cohort analysis of 12 national surveys collected between 1995 and 2019, we show that this age gap is largely a function of a long-term generational decline in support for democracy, with little evidence of an independent life-cycle effect apparent. The combination of generational decline without a positive and counterbalancing life-cycle effect offers a sober prognosis of how support for democracy in the United States might look in the future.

The commitment of the people of the United States to a democratic system, long taken for granted,^1^ is now in doubt. A growing body of research has demonstrated their shaky support for democracy when this is understood as support for *concrete* democratic norms or institutions or a preference for pro- versus antidemocratic political candidates (e.g., Bartels 2020; Graham and Svolik 2020; Gibson 2021; Simonovits, McCoy, and Littvay 2022). There is, however, considerable evidence now that Americans’ commitment to democracy *even in the abstract* is also in decline. As Voeten puts it, “the United States is an example of a country where support for democracy has gone down while alternatives have become more acceptable” (Voeten 2017, p. 3; c.f. Drutman, Goldman, and Diamond 2020).

These trends can be seen in figure 1, which reports longitudinal estimates of support for democracy in the United States. The composite measure (main panel) is obtained from Claassen’s (2020) dynamic Bayesian estimates of democratic mood across multiple countries and years. Rather than using survey questions about respondents’ “satisfaction with democracy,” closer to Easton’s conceptualization of *specific* support (Easton 1975, p. 437), “democratic mood” is based on widely used questions gauging the desirability of democracy, its comparison to undemocratic alternatives, or evaluations of the latter (see, e.g., Norris 2011). In this way, it captures diffuse support for democracy as a political regime. Although the credible intervals overlap, Claassen’s estimate of democratic mood in the United States fell from well above the global average (zero) of 141 countries in the world in 1995 to close to that global average in 2020. In contrast, there was no commensurate decline in a group of comparator nations—the other six members of the G7 group of high-income democracies.

**Figure 1.**
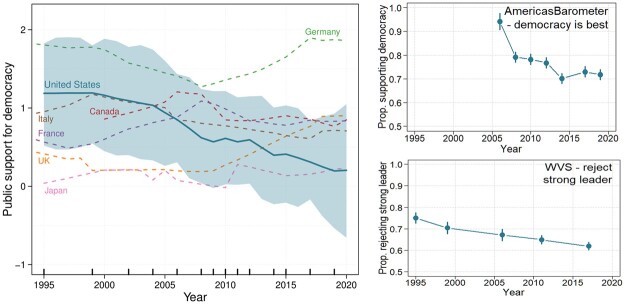
Declining public support for democracy in the United States, 1995 to 2020. The figure on the left shows Bayesian estimates of latent public support for democracy in the United States and the other G7 nations, based on Claassen (2020). Data are standardized such that the mean level of support for democracy across 141 countries and 33 years equals zero and the standard deviation equals one. The US estimates rely on survey data from the World Values Survey, AmericasBarometer, Pew Research, and the Comparative Study of Electoral Systems. Years in which survey data were collected are shown by the rug plot on the x-axis. The figures on the right show the proportions of US residents offering support for democracy in response to two particular questions routinely fielded by the World Values Survey (top) and AmericasBarometer (bottom) surveys. For the purposes of this figure, rejecting a strong leader is defined as selecting the “fairly bad” or “very bad” response options; supporting democracy is defined as selecting response options 5 through 7. See table 1 for question wordings and response options. See Supplementary Material figure S1 for more longitudinal estimates of specific items.

These trends remain visible when one examines specific survey questions: whereas 75 percent of respondents in the United States rejected a system of government with a “strong leader who does not have to bother with Congress and elections” in 1995, only 62 percent did so in 2017 (figure 1; upper-right plot). And, while 94 percent of Americans agreed that democracy “is better than any other form of government” in 2006, only 71 percent continued to do so in 2019 (figure 1; lower-right plot). In sum, while majorities remain supportive of democracy (Drutman, Goldman, and Diamond 2020), it is clear that support has declined substantially over the past few decades. To the extent that dwindling democratic support encourages antidemocratic strategies by political elites (Seligson and Booth 2009) or increases the electoral prospects of authoritarian leaders or antisystem parties (Mattes 2018; Cohen et al. 2022; Lewandowsky and Jankowski 2022), such a trend is troubling news for democracy in the United States.

One of the most concerning aspects of this ebbing attachment to democracy is a marked age gap, with younger Americans more hesitant about democracy and more supportive of nondemocratic alternatives (Foa and Mounk 2016; Norris 2017; Malka et al. 2020; Gibson 2021). However, it remains unclear how we should interpret this age gap. Is it a life-cycle effect, whereby younger citizens have always been more skeptical about democracy than their older fellow citizens (e.g., Norris 2017)? Or is it an indicator of generational change, in which the younger generations have lost support for democracy (Foa and Mounk 2016)? The consequences could not be more starkly different: if a life-cycle effect, youthful detachment will naturally transform into system support with the passage of time; if a cohort effect, it is political culture in the United States that could be transformed, as older generations who are more supportive of democracy are replaced by younger generations who are more open to authoritarian governance.

To separate the effects of aging (i.e., life-cycle effects) from the effects of generational change (i.e., cohort effects), we turn, in this paper, to age-period-cohort analyses. While such analyses of European support for democracy have been conducted (where little to no cohort effects are uncovered; Wuttke et al. 2020), the US case has been neglected, despite accumulating evidence of diminishing American support for democracy (e.g., figure 1). Using Bayesian Generalized Additive age-period-cohort models, and data from 12 national surveys collected between 1995 and 2019, we find little evidence of a life-cycle effect. Instead, we show that support for democracy and rejection of authoritarian rule has decreased generationally in the United States. This suggests that the decline in support for democracy is not easily reversible.

## Data and Methods

We collect survey measures of support for democracy from two public opinion projects: the World Values Survey (WVS) and the AmericasBarometer (AB). Each has polled nationally representative samples of Americans multiple times across a decade or more. Between 1995 and 2017, in five separate surveys, the WVS fielded five questions asking US respondents about their support for democracy or rejection of undemocratic rule. And between 2006 and 2019, in seven surveys, the AB included a question asking US respondents about their support for democracy. These survey data are described in table 1.

**Table 1.** Survey measures of US support for democracy.

World Values Survey (WVS)		
Question wording	Response set	Years fielded
I’m going to describe various types of political systems and ask what you think about each as a way of governing this country. For each one, would you say it is a very good, fairly good, fairly bad or very bad way of governing this country?	1: very good 2: fairly good 3: fairly bad 4: very bad	1995, 1999, 2006, 2011 & 2017
- Having a democratic political system		
- Having the army rule		
- Having a strong leader who does not have to bother with Congress and elections		
- Having experts, not government, make decisions according to what they think is best for the country		
Democracy may have problems but it’s better than any other form of government	1: agree strongly 2: agree 3: disagree 4: strongly disagree	1995 & 1999

AmericasBarometer		
Question wording	Response set	Years fielded
Changing the subject again, democracy may have problems, but it is better than any other form of government. To what extent do you agree or disagree with this statement?	1: strongly disagree 2-6: unlabelled 7: strongly agree	2006, 2008, 2010, 2012, 2014, 2017 & 2019

*Note*: Pooled number of respondents available: 8,819 (WVS); 9,609 (AmericasBarometer).

We analyze each dataset separately, testing if our results remain robust across the two. Since there are five “support for democracy” items included in the WVS, we first estimate a scale using a graded response model, a form of item-response theoretic (IRT) model.^2^ The single support for democracy item included in the AB is treated as ordinal due to the use of a seven-point response set.

It is well known that linear age, period, and cohort effects are not separately identifiable even when one has access to data that includes different age groups and is gathered over a period of many years (see Fosse and Winship 2019 for a review). For example, when it comes to survey data, a respondent’s age is nothing more than their year of birth (i.e., their cohort) and the date when they completed the survey (i.e., the period). Researchers instead estimate linear combinations of these effects (e.g., the overall trend, period + cohort effects) or treat one or more of these effects as nonlinear. Two specific models have been favored for this task by researchers in political science: hierarchical age-period-cohort models (HAPCMs; e.g., Schwadel and Garneau 2014; Smets and Neundorf 2014) and generalized additive models (GAMs; e.g., Grasso 2014; Jiang and Carriere 2014; Wuttke et al. 2020). The essence of each approach is to separate the effects of interest (e.g., age, cohorts) into both a linear and a nonlinear component. The latter can be identified for age groups, periods, and cohorts even though only two of their linear effects are identifiable. The HAPCM accomplishes this by modeling cohorts (and often also age groups and periods) as random effects. The GAM approach models cohorts (or year of birth) using cubic spline functions. We prefer the latter, as it avoids having to rely on arbitrary cohort or generational definitions (Jiang and Carriere 2014). Our HAPCM results are similar, however, and are included in the Supplementary Material.

Specifically, we model respondents’ support for democracy as a function of their cohort (using cubic splines for year of birth), age (using fixed effects for age groups 18–29, 30–44, 45–59, and over 60), and the period of the survey (using fixed effects for survey year). We run these models both with and without additional control variables. On the one hand, any generational patterns in support for democracy are of descriptive interest: they are noteworthy regardless of whether generation remains significant when controlling for other demographic factors. On the other hand, it is also worth investigating whether any generational effects are robust to including controls, which helps establish whether declining support for democracy in the United States is best characterized as a generational problem, or, for example, a class one. As control variables, we include: Republican and Democratic identity (vs. independent), having a college degree, identifying as female, identifying as white, living in the South, and self-reported income (standardized within each survey wave). The single support for democracy item in the AmericasBarometer data is modeled as an ordinal variable using ordered logit GAMs. The IRT measure of support for democracy obtained from the WVS data is treated as a continuous variable and modeled using linear GAMs. All analyses are weighted using the weights included in the WVS and AmericasBarometer datasets.^3^ Respondents with missing values are dropped via listwise deletion.

We estimate all models using Bayesian Markov Chain Monte Carlo (MCMC) methods, which allows more complex models to be fit and more accurate variance estimates to be obtained than the corresponding restricted maximum likelihood methods. We use the brms package, which calls the Stan modeling platform from R. Weakly informative priors are used. All models show convergence, as indicated by trace plots R-hat statistics of close to 1 (specifically, less than 1.02; see Supplementary Material).

## Results

One conjecture about the decline of democratic support in the United States is that American millennials (by convention, those born from the early 1980s until the mid-1990s) have particularly low levels of democratic support. The main rationale is that this cohort represents the first group of Americans whose crucial formative years were spent in the post–Cold War world, a context where threats to democracy became less plausible and vivid than for previous generations (Foa and Mounk 2016 and 2019). Furthermore, this millennial generation is also argued to have grown into adulthood under deteriorating economic conditions (Denemark et al. 2016, p. 184), including growing income inequality and stagnating incomes for the lower and middle classes (Foa and Mounk 2019, p. 1021). These conditions may have rendered this cohort particularly open to contemplating alternatives to the democratic status quo, in contrast with previous generations.

**Figure 2.**
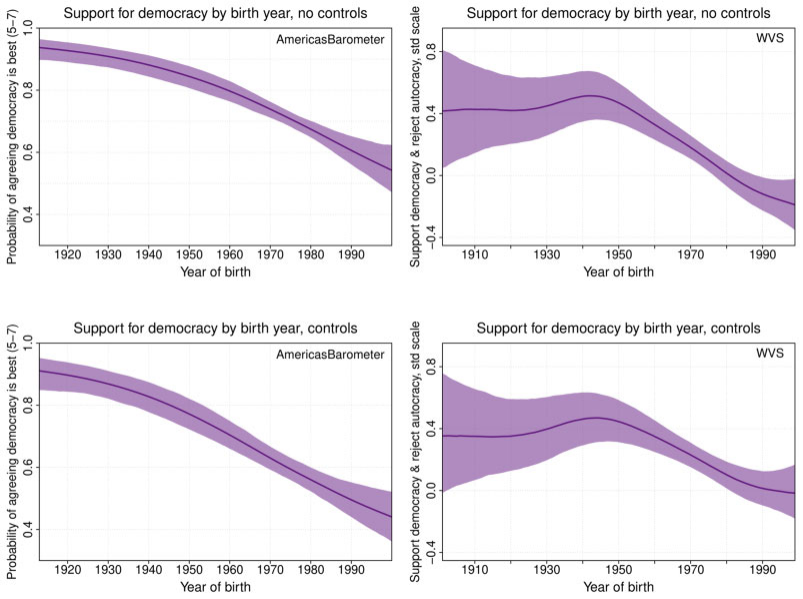
Generational effects. AmericasBarometer estimates (left) are from a single item; ordered logit GAM used. “Agreeing democracy is best” is defined for the purposes of this figure as selecting response category 5 or higher; the underlying model treats the outcome as ordinal, however. WVS estimates (right) use a five-item scale; linear GAM used. Demographic controls including for models in bottom row and excluded in top row.

In figure 2, we examine this conjecture by presenting the smoothed GAM estimates of support for democracy across year of birth (see also the tables of parameter estimates—tables 2 and 3). The AmericasBarometer results (left) show that the more recent the year of birth, the lower the agreement with the notion that “democracy may have problems, but it is better than any other form of government.” Respondents born before and during the Second World War (i.e., the “Silent Generation”) are more than 80 percent likely to express support for democracy in this way, while respondents born in the 1970s and 1980s are less than 70 percent likely to support democracy. The generational effects are even more pronounced when demographic variables are omitted from the GAM (top left figure).

**Table 2.** Parameter estimates, AmericasBarometer GAM.

	Model 1		Model 2	
	Parameter estimate	Standard error	Parameter estimate	Standard error
Birth year spline variance parameter	0.64	0.05	0.52	0.36
Birth year fixed effect	−3.11	1.19	−2.71	1.33
Year: 2008	−1.03	0.10	−1.02	0.15
Year: 2010	−1.10	0.10	−1.04	0.14
Year: 2012	−1.15	0.11	−1.09	0.14
Year: 2014	−1.43	0.11	−1.33	0.15
Year: 2017	−1.23	0.11	−1.15	0.15
Year: 2019	−1.13	0.12	−1.09	0.15
Age: 30−44	−0.13	0.09	−0.18	0.09
Age: 45−59	−0.19	0.15	−0.28	0.15
Age: 60+	−0.11	0.19	−0.22	0.20
Republican			0.71	0.05
Democrat			0.41	0.04
Has degree			0.29	0.05
Female			−0.35	0.04
White			0.08	0.04
Income			0.20	0.02
South			0.04	0.04
Cutpoint1	−4.96	0.14	−4.77	0.18
Cutpoint2	−4.37	0.13	−4.19	0.17
Cutpoint3	−3.64	0.13	−3.45	0.17
Cutpoint4	−2.50	0.13	−2.26	0.17
Cutpoint5	−1.71	0.13	−1.43	0.17
Cutpoint6	−0.78	0.13	−0.45	0.17

*N*	9,584		8,902	

*Note:* Results for World Values Survey Generalized Additive Model estimated using Bayesian MCMC methods, as implemented in the brms library for R. Three chains were run in parallel for 2,000 iterations, with the first 1,000 of these being dedicated to warmup of the MCMC algorithm. Convergence is diagnosed by examination of R-hat statistics and posterior predictive plots. “Parameter estimates” are the mean of the posterior distributions for each parameter across the 3,000 post-warmup iterations (i.e., 1,000 × 3 chains); “standard errors” are the standard deviation of these parameter posterior distributions.

**Table 3.** Parameter estimates, World Values Survey GAM.

	Model 1		Model 2	
	Parameter estimate	Standard error	Parameter estimate	Standard error
Birth year spline variance parameter	0.73	0.35	0.64	0.33
Birth year fixed effect	−0.43	0.72	−0.20	0.71
Year: 1999	−0.30	0.04	−0.33	0.04
Year: 2006	−0.32	0.04	−0.32	0.05
Year: 2011	−0.34	0.05	−0.36	0.05
Year: 2017	−0.34	0.06	−0.47	0.06
Age: 30−44	−0.02	0.04	−0.04	0.05
Age: 45−59	−0.05	0.07	0.01	0.08
Age: 60+	0.02	0.11	0.14	0.11
Republican			0.08	0.03
Democrat			0.18	0.03
Has degree			0.43	0.02
Female			−0.15	0.02
White			0.25	0.03
South			−0.10	0.02
Income			0.01	0.01
Intercept	0.25	0.04	−0.07	0.05
Residual standard deviation	0.97	0.01	0.94	0.01

*N*	8,797		7,474	

*Note*: Results for World Values Survey Generalized Additive Model estimated using Bayesian MCMC methods, as implemented in the brms library for R. Three chains were run in parallel for 2,000 iterations, with the first 1,000 of these being dedicated to warmup of the MCMC algorithm. Convergence is diagnosed by examination of R-hat statistics and posterior predictive plots. “Parameter estimates” are the mean of the posterior distributions for each parameter across the 3,000 post-warmup iterations (i.e., 1,000 × 3 chains); “standard errors” are the standard deviation of these parameter posterior distributions.

The WVS results (figure 2, on the right) show, on their face, a slightly different result. Support for democracy remains stable for the 1910s to 1940s birth cohorts, and subsequently reaches its highest level for respondents born around the time of the Second World War. However, support then follows the same precipitous decline as seen in the AmericasBarometer data. As we reach those born since the 1980s, support for democracy is more than half a standard deviation lower than its Second World War peak—net the effects of age, period, and demography.^4^

To be sure, for both the AB and the WVS data, estimates for the most extreme cohorts must be taken with caution, given potential biases caused by lack of common support (i.e., no data for youthful respondents in the earliest cohorts or for elderly respondents in the latest cohorts). Still, the message common to both sets of results is that there has been a pattern of cohort decline in democratic support taking place at least since those born in the 1940s. Furthermore, both analyses also raise doubts about whether there might be something unique about the “millennial generation” in terms of its support for democracy, measured either using a single survey question (as in the AmericasBarometer data) or with a more nuanced measure encompassing rejection of autocratic forms of government (as in the WVS data). Instead, each cohort born after the 1940s has been less supportive of democracy than the one before. From this point of view, we have no evidence that millennials are anything other than at the tail end of a long-term trend of generational decline in democratic support.

**Figure 3.**
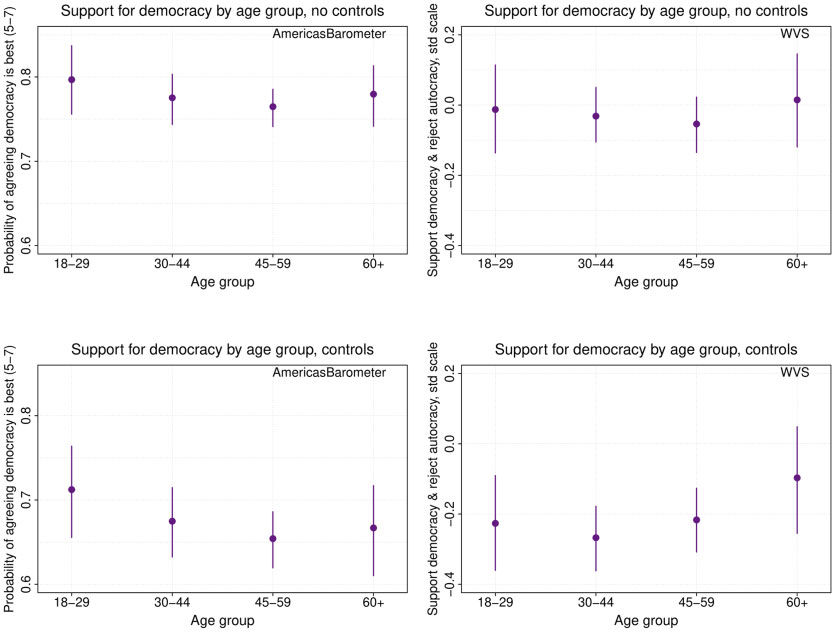
Age effects. Predicted effects of support for democracy by age group, with other variables set at means or modes. AmericasBarometer estimates (left) are based on an ordered logit GAM. WVS estimates (right) are based on a linear GAM.

Importantly, these results are obtained while simultaneously adjusting for (nonlinear) age effects. That younger Americans are less supportive of democracy than their parents could just be a function of a *life-cycle effect* (Alexander and Welzel 2017; Norris 2017), linked to the greater political indifference and inattention of younger citizens (Nemčok and Wass 2021), their lower experience with the functioning of a democratic regime (Sapiro 2004), or even the systematic descriptive and substantive underrepresentation of the young in most democracies (Curry and Haydon 2018; Sundström and Stockemer 2021). However, as we can see in figure 3, our age-period-cohort analysis shows there is not much of a life-cycle effect in democratic support in the United States accordingly, in contrast to the marked cohort effects we found before. There are no significant differences between the four age groups once cohort and period effects are considered (see also Supplementary Material figure S4 for similar results from HAPC models).

**Figure 4.**
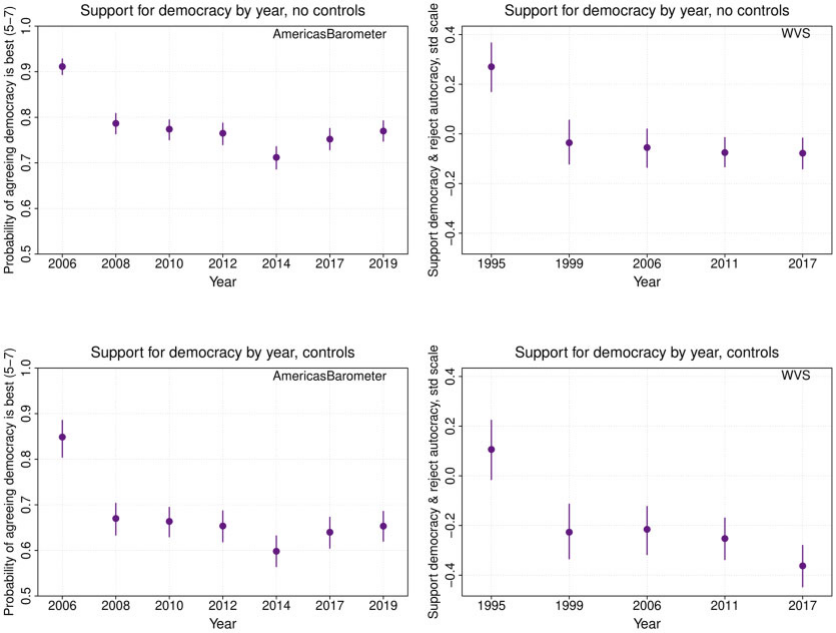
Period effects. Predicted effects of support for democracy by survey year, with other variables set at means or modes. AmericasBarometer estimates (left) are based on an ordered logit GAM. WVS estimates (right) are based on a linear GAM.

Finally, we also consider period effects, which are shown in figure 4. Period effects reflect the time-specific factors that have shaped national levels of support for democracy, including short-run events and long-run secular changes, but also survey-specific methodological factors. As can be seen in figure 4, we find higher support for democracy in the first wave of AmericasBarometer and WVS surveys, net the effects of age and generation (which may well be a methods effect relating to those first-wave surveys). There is otherwise little evidence of temporal variation, which suggests that the trend of declining support for democracy in the United States shown in figure 1 is largely a product of more supportive older generations being replaced by less supportive younger ones.

## Conclusions

Support for democracy in the United States is not as solid as it once seemed. While a growing number of authors have demonstrated weak public support for specific democratic institutions and norms or candidates committed to democracy, we show that diffuse support for a democratic regime in the abstract is weaker than it once was.

Existing research has found a marked effect of age on support for democracy in the United States, with younger citizens demonstrating significantly lower levels of democratic commitment. Some have attributed this age gap to a cohort effect, for which the generation of those born in the 1980s and 1990s is argued to be particularly responsible. Others have attributed this to a life-cycle effect, in which support for democracy is weaker for younger individuals but is later learned as one matures and becomes socialized into the political system.

Using age-period-cohort analysis, we find no evidence for such a life-cycle effect. Furthermore, we demonstrate that generational decline in support for democracy did not start with the cohort of those who spent their crucial formative years in a post–Cold War context. Instead, American support for democracy has been weakening in one cohort after the next at least since the Second World War. This trend echoes the findings of age-period-cohort analyses of political tolerance (Schwadel and Garneau 2014) and civic participation (Caren, Ghoshal, and Ribas 2011) in the United States. But it diverges from the findings of a previous age-period-cohort analysis of support for democracy in Europe, where “generational disparities are narrow in most cases …, and members of this [most recent] cohort remain committed to democracy as a viable system of government” (Wuttke et al. 2020, p. 10).

The combination of generational decline and the lack of a positive life-cycle effect offers a sobering prognosis of how support for democracy in the United States might look in the future. Younger generations already have substantially lower democratic support than the older generations whom they will replace. Without a positive life-cycle effect to counter this intergenerational replacement, the United States faces a continued decline in public commitment to a democratic system of government. Such decline justifies concerns with the future endurance of democratic institutions in the face of potential political, social, and economic crises and with the availability of the American public to shut out leaders and movements set on undermining liberal democracy.

## Supplementary Material

nfad039_Supplementary_Data

## Data Availability

Replication data and documentation are available at https://doi.org/10.7910/DVN/HVYTNM.

## Footnotes

1
Graham and Svolik (2020, p. 392) make this point vividly by quoting Robert Dahl: “It is nearly impossible to find an American who says that he is opposed to democracy or favors some alternative” (Dahl 1966, p. 40).

2 Creating a scale out of all available items reduces measurement error and is therefore preferable to examining each item separately (see Berinsky 2017 for a discussion). The additive scale of the five WVS items shows adequate interitem reliability (Cronbach’s alpha = 0.67, mean correlation = 0.28).

3 It is not possible, using the information made available in the public WVS and AmericasBarometer datasets, to account for design effects. Such effects are not included in the estimates of uncertainty we present in this paper.

4 See the Supplementary Material for additional analyses, including HAPC models (Supplementary Material figure S3) as well as GAM models employing more restricted three- and four-item scales from the WVS data (Supplementary Material figure S6). Results are fundamentally similar.
