# Supplementary material for: Public Support for Democracy in the United States Has Declined Generationally
Source: Public Opin Q. 2023 Sep 15;87(3):719–32. doi: 10.1093/poq/nfad039 (PMC10662659; doi:10.1093/poq/nfad039)
Supplement: nfad039_Supplementary_Data [file nfad039_supplementary_data.pdf]

Supplementary Material

Public Support for Democracy in the United States Has Declined Generationally

Christopher Claassen
School of Social and Political Sciences,
University of Glasgow
christopher.claassen@glasgow.ac.uk

Pedro C. Magalhães
Institute of Social Sciences,
University of Lisbon
pedro.magalhaes@ics.ulisboa.pt

Table S1: Metholodological Details of Survey Fieldwork

World Values Survey (United States samples)

1995: https://www.worldvaluessurvey.org/WVSDocumentationWV3.jsp?COUNTRY=1283
1999: https://www.worldvaluessurvey.org/WVSDocumentationWV4.jsp?COUNTRY=507
2006: https://www.worldvaluessurvey.org/WVSDocumentationWV5.jsp?COUNTRY=467
2011: https://www.worldvaluessurvey.org/WVSDocumentationWV6.jsp?COUNTRY=341
2017: https://www.worldvaluessurvey.org/WVSDocumentationWV7.jsp

AmericasBarometer (United States samples)

2006: http://www.vanderbilt.edu/lapop/usa/2006-techinfo.pdf
2008: http://www.vanderbilt.edu/lapop/usa/2008-techinfo.pdf
2010: https://www.vanderbilt.edu/lapop/ab2010/2010-Design-Effects.pdf
2012: https://www.vanderbilt.edu/lapop/ab2012/AB-2012-Tech-Info-12.18.12.pdf
2014: http://www.vanderbilt.edu/lapop/ab2014/AB-2014-Tech-Info-112114-W.pdf
2017:
https://www.vanderbilt.edu/lapop/usa/United_States_AmericasBarometer_Tech_Info_2016_17_W_092217.pdf
2019: https://www.vanderbilt.edu/lapop/usa/US_AmericasBarometer_2018-19_Technical_Report_W_101019.pdf

Figure S1: Rejection of Autocracy / Support for Democracy, World Values Survey Questions, 1995-2017

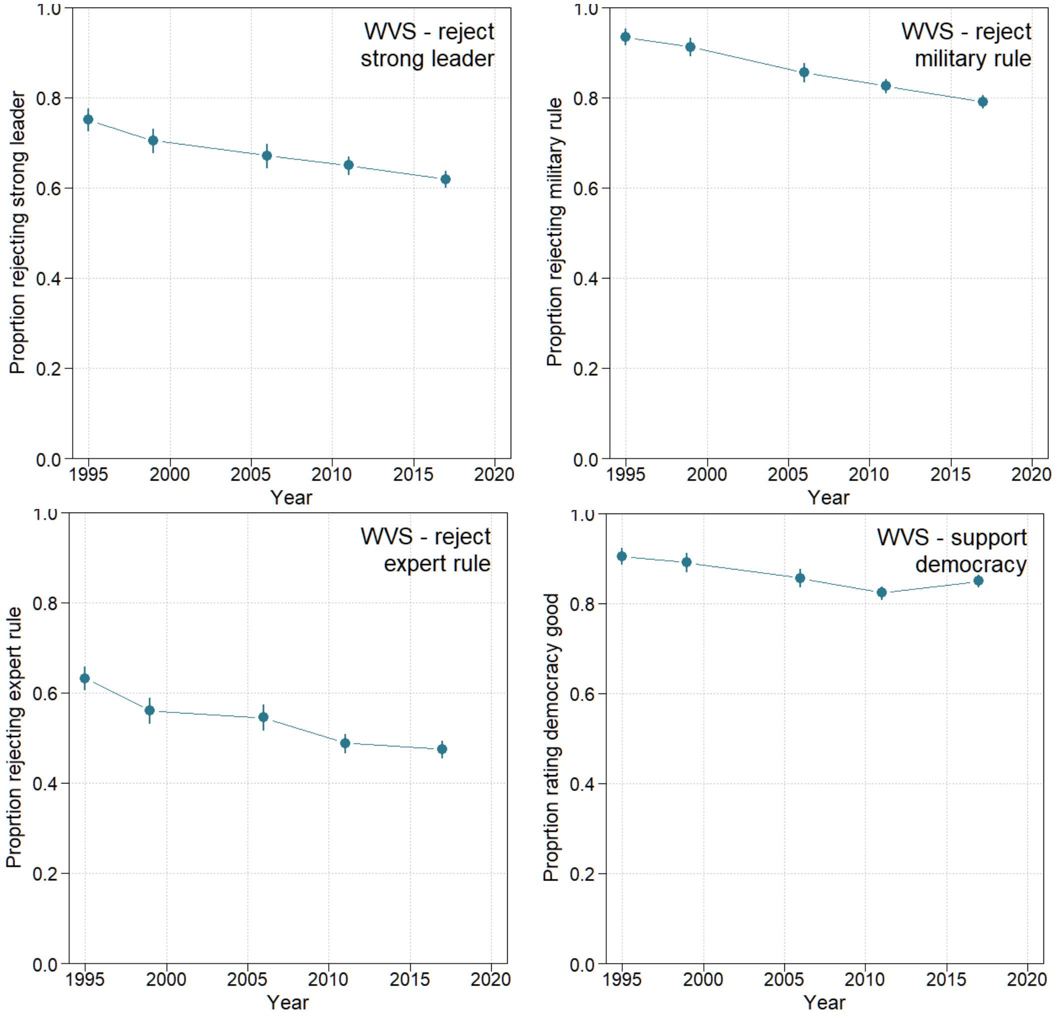

Notes. Each figure shows the proportion of each WVS sample supporting democracy or rejecting autocracy, broken down by each of the four main questions from the WVS. The "democracy is best" question is omitted as it is only fielded in 1995 and 1999. For the purposes of this figure, rejecting autocracy is defined as rating a particular regime as "fairly bad" or "very bad"; supporting democracy is defined as evaluating a democratic system as "fairly good" or "very good."

Figure S2: Generational Effects, Bivariate

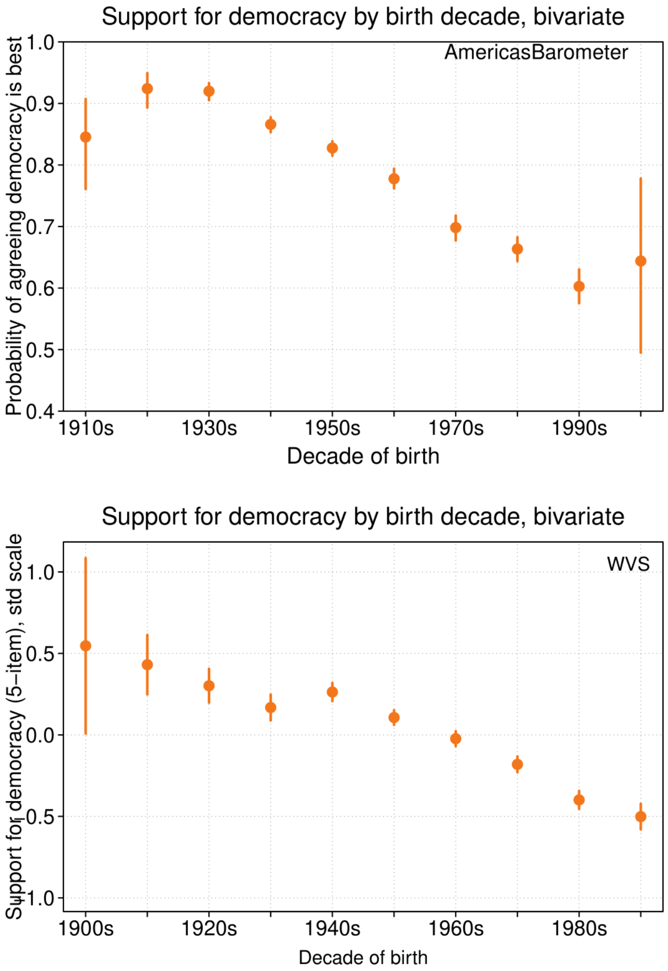

Notes. These figures show the average level of support for democracy in the pooled AmericasBarometer (top) and World Values Survey (bottom) samples for each birth cohort. AmericasBarometer results are predicted probabilities based on an ordered logit model with birth decade as the only predictor; WVS results are obtained by applying a linear model to the five-item scale, with birth decade as the only predictor.

Table S2: Parameter Estimates, AmericasBarometer HAPCMs

	Parameter estimate	Standard error	Parameter estimate	Standard error
Cutpoint1	-3.60	.36	-3.41	.36
Cutpoint2	-3.02	.36	-2.82	.36
Cutpoint3	-2.29	.35	-2.08	.35
Cutpoint4	-1.15	.35	-.89	.35
Cutpoint5	-.36	.35	-.07	.35
Cutpoint6	.57	.36	.91	.35
Republican			.71	.05
Democrat			.41	.04
Has degree			.29	.05
Female			-.34	.04
White			.09	.04
Income			.20	.02
South			.04	.04
Age group standard deviation	.26	.23	.23	.22
Birth decade standard deviation	.63	.22	.65	.22
Survey year standard deviation	.60	.22	.59	.23
N	9584		8902	

Notes: Results for AmericasBarometer Hierarchical Age-Period-Cohort Models estimated using Bayesian MCMC methods, as implemented in the brms() library for R. Three chains were run in parallel for 2,000 iterations, with the first 1,000 of these being dedicated to warmup of the MCMC algorithm. Age (4 groups), Year (5 groups) and birth decade (10 groups) are specified as varying / random effects. The outcome is specified as ordinal and a logit link function is used. “Parameter estimates” are the mean of the posterior distributions for each parameter across the 3,000 post-warmup iterations (i.e., 1,000 × 3 chains); “standard errors” are the standard deviation of these parameter posterior distributions.

Table S3: Parameter Estimates, World Values Survey HAPCM

	Parameter estimate	Standard error	Parameter estimate	Standard error
Intercept	-.03	.01	-.35	.23
Republican			.09	.03
Democrat			.18	.03
Female			-.15	.02
White			.26	.03
Income			.01	.01
Has degree			.43	.02
South			-.10	.02
Residual standard deviation	.97	.01	.94	.01
Age group standard deviation	.26	.19	.29	.19
Birth decade standard deviation	.13	.05	.10	.04
Survey year standard deviation	.29	.16	.31	.16
N	8797		7474	

Notes: Results for World Values Survey Hierarchical Age-Period-Cohort Models estimated using Bayesian MCMC methods, as implemented in the brms() library for R. Three chains were run in parallel for 2,000 iterations, with the first 1,000 of these being dedicated to warmup of the MCMC algorithm. Age (4 groups), Year (5 groups) and birth decade (10 groups) are specified as varying / random effects. "Parameter estimates" are the mean of the posterior distributions for each parameter across the 3,000 post-warmup iterations (i.e., 1,000 × 3 chains); "standard errors" are the standard deviation of these parameter posterior distributions.

Figure S3: Generational Effects, Hierarchical Age-Period-Cohort Models

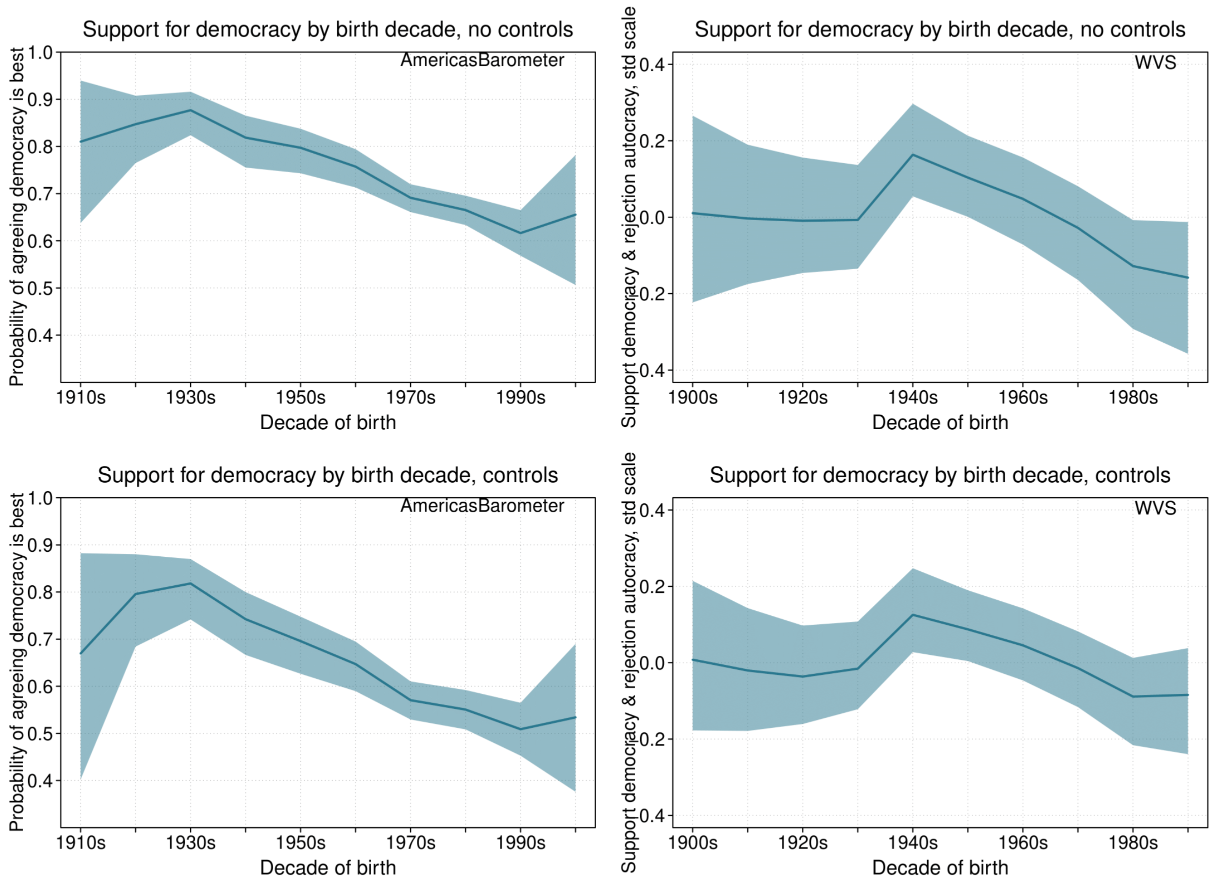

Notes: Estimates obtained from HAPC models, estimated using Bayesian MCMC methods. A hierarchical ordered logit specification is used to model the single item fielded by the AmericasBarometer (left); a hierarchical linear specification is used to model the support for democracy scale constructed using the five items fielded by the World Values Survey (right). Demographic controls including for models in bottom row and excluded in top row.

Figure S4: Age Effects, Hierarchical Age-Period-Cohort Models

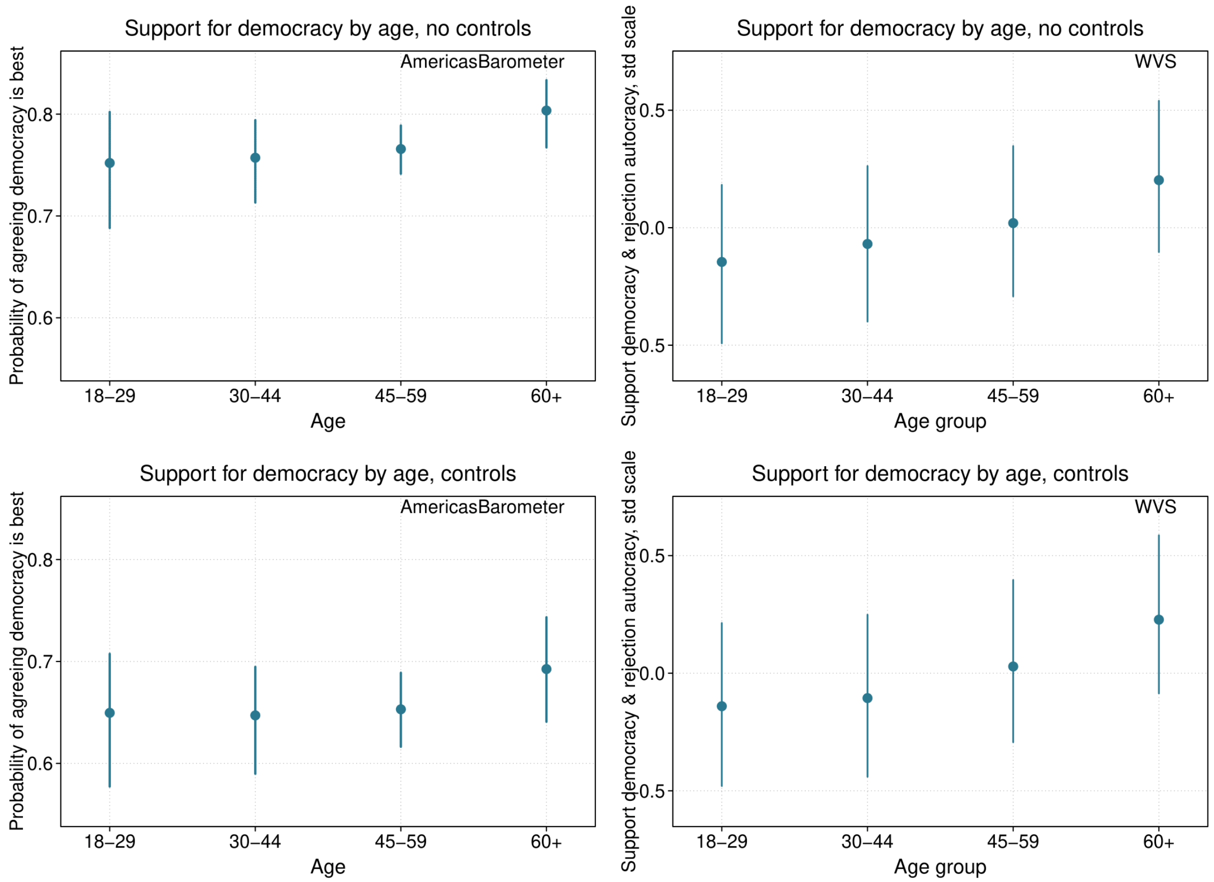

Notes: Estimates obtained from HAPC models, estimated using Bayesian MCMC methods. A hierarchical ordered logit specification is used to model the single item fielded by the AmericasBarometer (left); a hierarchical linear specification is used to model the support for democracy scale constructed using the five items fielded by the World Values Survey (right). Demographic controls including for models in bottom row and excluded in top row.

Figure S5: Period Effects, Hierarchical Age-Period-Cohort Models

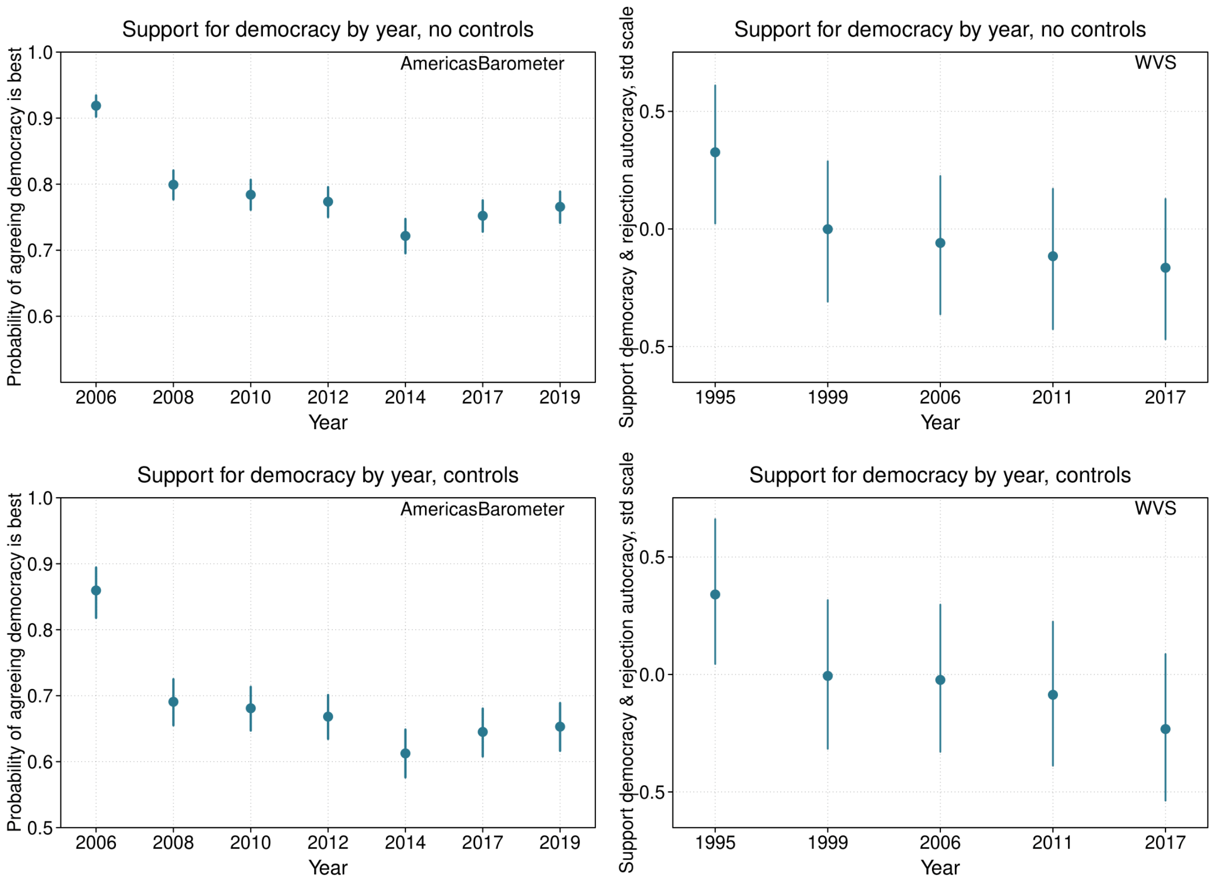

Notes: Estimates obtained from HAPC models, estimated using Bayesian MCMC methods. A hierarchical ordered logit specification is used to model the single item fielded by the AmericasBarometer (left); a hierarchical linear specification is used to model the support for democracy scale constructed using the five items fielded by the World Values Survey (right). Demographic controls including for models in bottom row and excluded in top row.

Figure S6: GAM Estimates Using Alternative WVS Support for Democracy Scales

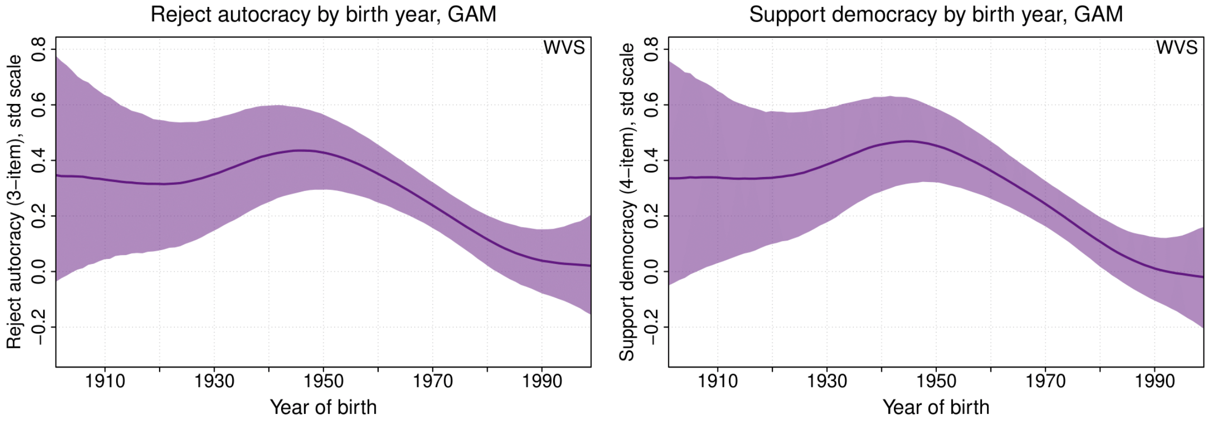

Notes: GAM estimates obtained using a (left) three-item scale comprising the three "evaluate authoritarian rule" questions from the WVS and (right), a four-item scale comprising the three "evaluate authoritarian rule" questions and the evaluate democracy question. Control variables are included for both models.

Figure S7: MCMC Convergence: AmericasBarometer GAMs

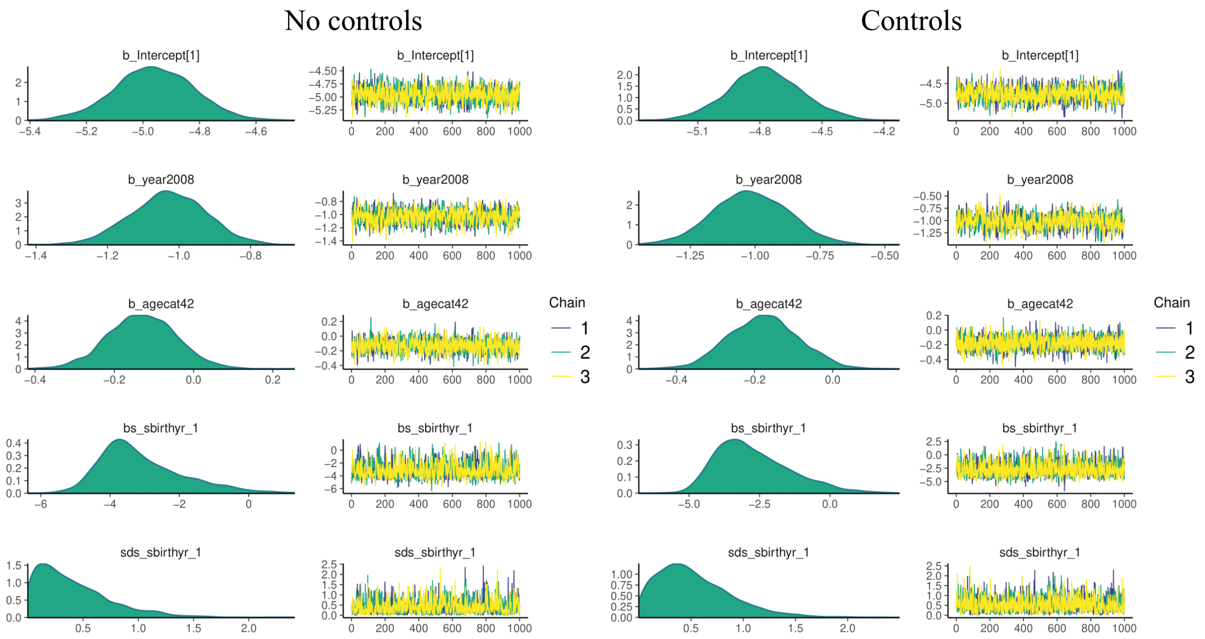

Notes: Trace and density plots of selected parameters for Bayesian GAMs, AmericasBarometer data. Model without controls on left; with, on right.

Figure S8: MCMC Convergence: WVS GAMs

No controls | Controls

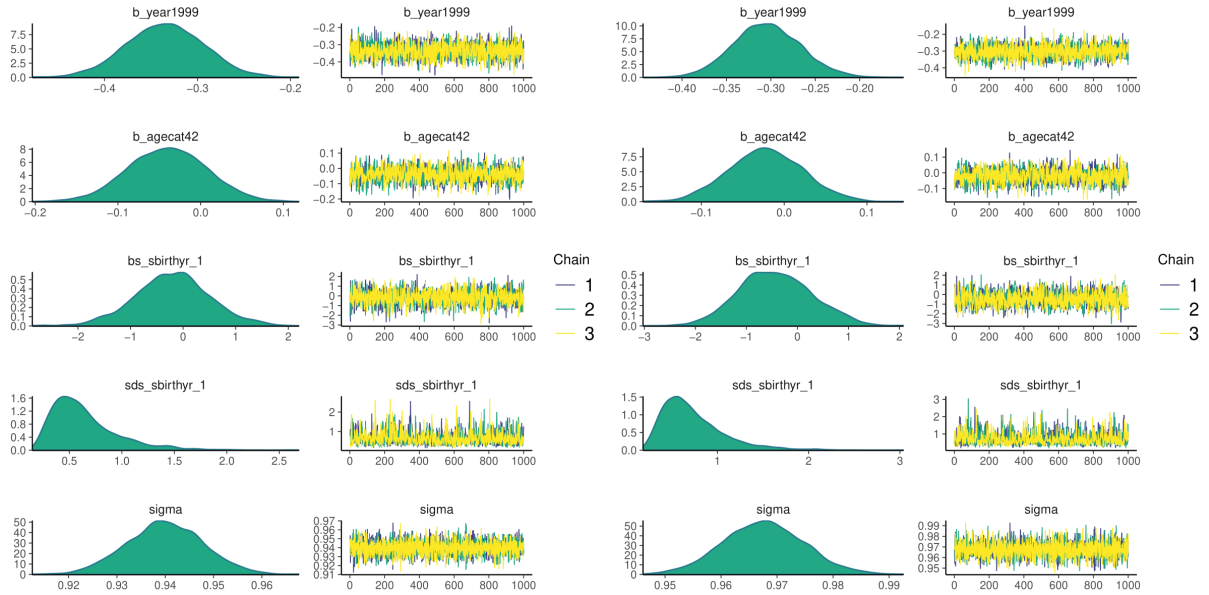

Notes: Trace and density plots of selected parameters for Bayesian GAMs, WVS data. Model without controls on left; with, on right.

Figure S9: MCMC Convergence: AmericasBarometer HAPCMs

No controls | Controls

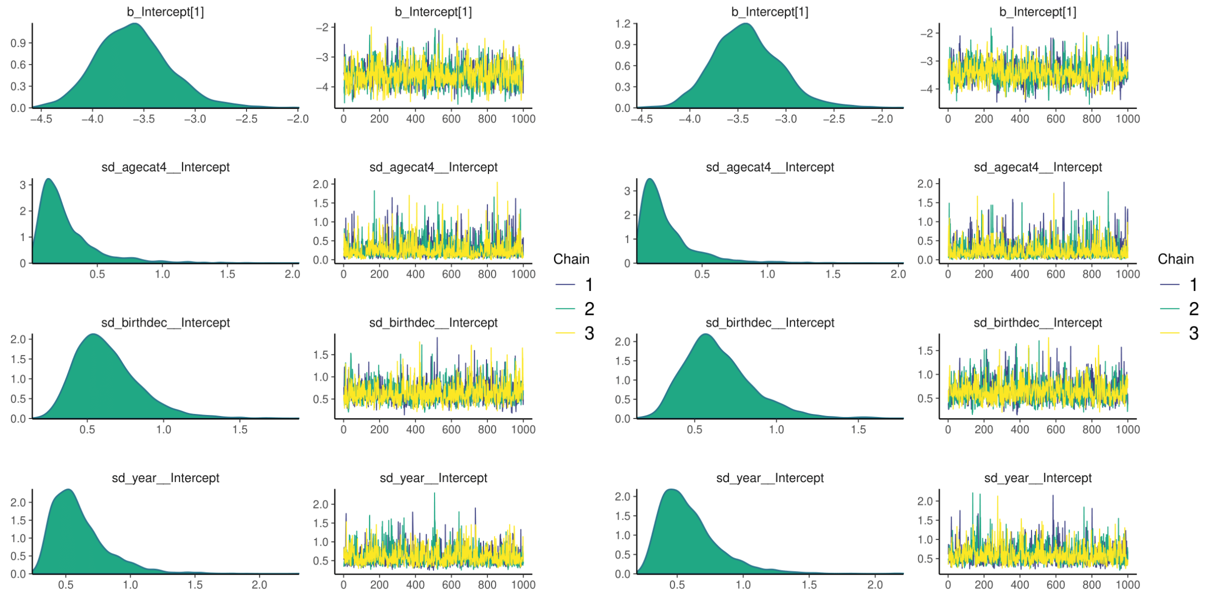

Notes: Trace and density plots of selected parameters for Bayesian HAPCMs, AmericasBarometer data. Model without controls on left; with, on right.

Figure S10: MCMC Convergence: WVS HAPCMs

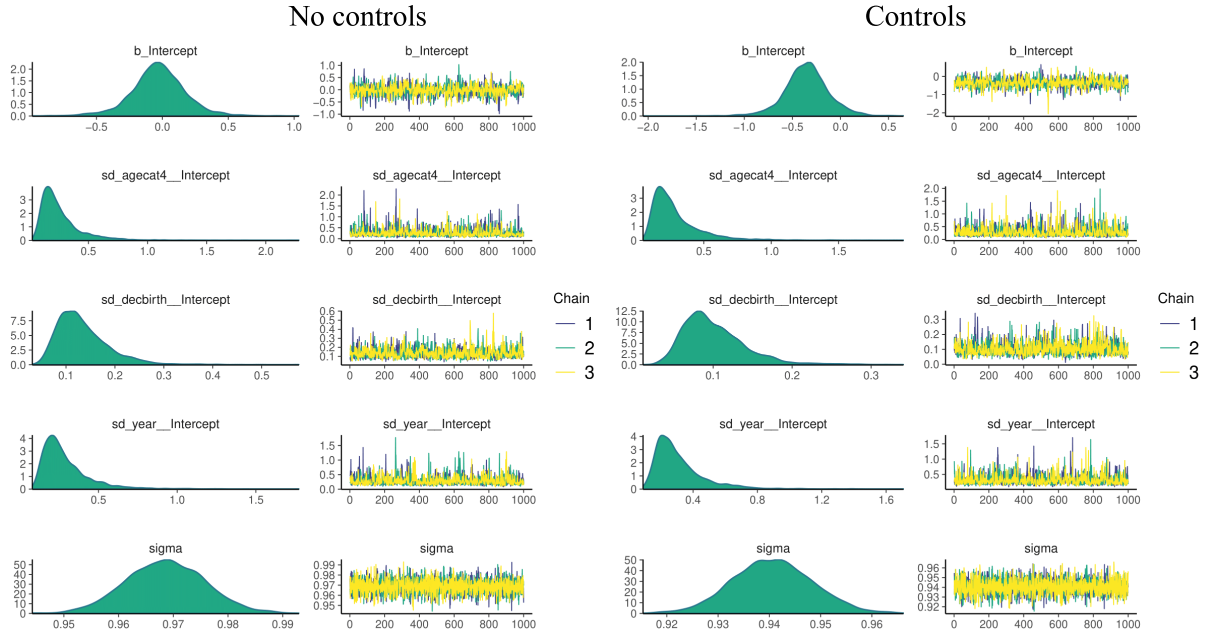

Notes: Trace and density plots of selected parameters for Bayesian HAPCMs, WVS data. Model without controls on left; with, on right.
